# New Population and Phylogenetic Features of the Internal Variation within Mitochondrial DNA Macro-Haplogroup R0

**DOI:** 10.1371/journal.pone.0005112

**Published:** 2009-04-02

**Authors:** Vanesa Álvarez-Iglesias, Ana Mosquera-Miguel, Maria Cerezo, Beatriz Quintáns, Maria Teresa Zarrabeitia, Ivon Cuscó, Maria Victoria Lareu, Óscar García, Luis Pérez-Jurado, Ángel Carracedo, Antonio Salas

**Affiliations:** 1 Unidade de Xenética, Instituto de Medicina Legal and Departamento de Anatomía Patolóxica y Ciencias Forenses, Facultade de Medicina, Universidade de Santiago de Compostela, Galicia, Spain; 2 Fundación Pública Galega de Medicina Xenómica (FPGMX), and Ciber de enfermedades raras (CIBERER), Hospital Clínico Universitario, Universidade de Santiago de Compostela, Galicia, Spain; 3 Medicina Legal, Universidad de Cantabria, Santander, Spain; 4 Unidad de Genética, Universitat Pompeu Fabra, and U735 CIBER de enfermedades raras (CIBERER), Barcelona, Spain; 5 Laboratorio de la Ertzaintza, Bilbao, Spain; 6 Programa de Medicina Molecular y Genética, Hospital Universitari Vall d'Hebron, Barcelona, Spain; University of Glasgow, United Kingdom

## Abstract

**Background:**

R0 embraces the most common mitochondrial DNA (mtDNA) lineage in West Eurasia, namely, haplogroup H (∼40%). R0 sub-lineages are badly defined in the control region and therefore, the analysis of diagnostic coding region polymorphisms is needed in order to gain resolution in population and medical studies.

**Methodology/Principal Findings:**

We sequenced the first hypervariable segment (HVS-I) of 518 individuals from different North Iberian regions. The mtDNAs belonging to R0 (∼57%) were further genotyped for a set of 71 coding region SNPs characterizing major and minor branches of R0. We found that the North Iberian Peninsula shows moderate levels of population stratification; for instance, haplogroup V reaches the highest frequency in Cantabria (north-central Iberia), but lower in Galicia (northwest Iberia) and Catalonia (northeast Iberia). When compared to other European and Middle East populations, haplogroups H1, H3 and H5a show frequency peaks in the Franco-Cantabrian region, declining from West towards the East and South Europe. In addition, we have characterized, by way of complete genome sequencing, a new autochthonous clade of haplogroup H in the Basque country, named H2a5. Its coalescence age, 15.6±8 thousand years ago (kya), dates to the period immediately after the Last Glacial Maximum (LGM).

**Conclusions/Significance:**

In contrast to other H lineages that experienced re-expansion outside the Franco-Cantabrian refuge after the LGM (e.g. H1 and H3), H2a5 most likely remained confined to this area till present days.

## Introduction

Haplogroup R0, formerly known as pre-HV [1], is defined by the absence of transitions A73G and G11719A relative to haplogroup R. There is one main sub-branch of R0 defined by the lack of C14766T (haplogroup HV) and a minor branch known as R0a [2]. HV embraces the most frequent haplogroup in Europe (∼40%), namely, haplogroup H, which is defined by the lack of the characteristic transitions A2706G and C7028T. HV also contains some other less frequent clades, such as HV1, HV2, and specially HV0, where haplogroup V is nested. Most of the haplogroup H sub-lineages are most likely of Middle Eastern origin (as it is the case of the majority of the typical West European clades). Overall, R0 shows frequency patterns declining from West towards East and South Europe and Middle East [1], [3], [4].

By way of complete genome sequencing, Achilli et al. [1] identified numerous sub-branches of haplogroup H. These authors demonstrated for the first time that, although haplogroup H overall in Europe is rather uniform, the sub-clades H1 and H3 show frequency peaks centered in Iberia and surrounding areas. The phylogeographic distribution of these lineages and their coalescence ages (∼11 kya) lead these authors to conclude that H1 and H3 represent a signal of late-glacial expansion of hunter-gatherers that repopulated Central and Northern Europe from about 15,000 years ago, after the LGM. These patterns mirror those previously observed by the same authors for haplogroup V [5], which also shows a clear-cut clinal geographical distribution in Europe, with a peak in the Franco-Cantabrian area and coalescence ages ranging from 16.3±4.8 kya in West Europe to 8.5±2.3 kya in the East Europe. Therefore, the geographic and diversity patterns of these three lineages pointed to a re-colonization period of Europe from western refuge locations after the LGM period. Apart from adding substantially to the resolution of the haplogroup H phylogeny, another contemporary study [3] also showed that some lineages such as H1*, H1b, H1f, H2a, H3, H6a, H6b, and H8, have distinct phylogeographic patterns within Europe. The study by Brandstätter et al. [6] further contributed to the dissection of the phylogeny of haplogroup H, although with a more technical perspective. More recently, Roostalu et al. [4] studied the population stratification of haplogroup H sub-lineages in West Eurasia, with special focus to Near Eastern populations and the Caucasus. Again, the authors demonstrated that most of the haplogroup H lineages of present-day Near Eastern-Caucasus area expanded after the LGM and presumably before the Holocene. The study of Abu-Amero et al. [2] was also very useful in providing further resolution at the level of complete genome sequencing within R0a. The refined knowledge of the mtDNA phylogeny to the level of complete genomes opened the doors to a wide spectrum of different applications of medical and forensic interest; see also [7], [8], [9], [10].

On the other hand, the mtDNA phylogeny needs continuous updating in order to ease future population and phylogenetic studies (e.g. [11], [12]). Due to the growing interest of geneticists in unraveling the internal variation within haplogroup H, several conflicts have arisen in the phylogeny and nomenclature of R0 sub-clades. For example, the recent publication of Roostalu et al. [4] added new branches to the phylogeny of haplogroup H, but for instance, their label H19 was used to name a different branch in the contemporary study of Achilli et al. [1]. To give another example, based on complete genome sequencing, Behar et al. [13] referred to a new clade, R0a1, with three minor sub-clades (R0a1a, R0a1b, and R0a1c); however, they did not notice the contribution by Abu-Amero et al. [2], where new complete genomes and new sub-branches of R0a had been reported; thus, for instance, the R0a1 in Behar et al. [13] matched a branch previously coined (preHV)1b by Abu-Amero et al. [2] (therefore using also the old nomenclature; see [14]). Some of the problems related to R0a were mentioned in Brandstätter et al. [6] and Brandstätter et al. [15]; although many problems still remain (see below).

The goals of the present study are: i) provide new insights into the distribution and population variability of haplogroup H sub-lineages in North Iberia to a high level of phylogenetic resolution; ii) resolve the many existing conflicts in the nomenclature and phylogeny of R0 that nowadays represent a challenge for future inter-population studies; iii) refine the phylogeny of R0 by way of inspecting the existing mtDNA complete genomes (plus coding region segments) available in the literature and GenBank (>1,100); and iv) contribute to enrich the known phylogeny of haplogroup H at the level of complete genome sequencing, by characterizing a new autochthonous clade observed in the Basque Country, namely H2a5.

## Methods

### Samples

We have collected samples from three main North Iberia regions. A total of 282 healthy unrelated individuals were obtained from Galicia (northwest Iberia) (which is an independent sample to the one reported in [16], [17]). Three different locations were sampled in Cantabria (*N* = 135; North-Central Iberia), including 39 healthy unrelated individuals from Valle del Pas, 45 from Valle del Liébana, and 51 from Santander. Several individuals from Valle del Pas were previously reported for the HVS-I segment in Maca-Meyer et al. [18] . For most of the analysis, these three locations were lumped in a single group (Cantabria). A total of 101 individuals suffering autism were collected from Catalonia (northeast Iberia). Since mtDNA lineages do not play a role as medium to high penetrance factors in autism (which is likely to be a polygenic and multifactorial disease), this sample can be considered to represent (from a mtDNA point of view) a random sample from the region (a case-control association study performed by the authors adds support to this statement). Finally, eight samples carrying substitution C4592T in the sample set of 75 individuals from the Basque Country (bordering East Cantabria) screened in [19], and presumably belonging to a new minor clade of haplogroup H (here baptized as H2a5), were selected for complete genome sequencing.

Oral informed consent was required for the samples collected in Galicia and Cantabria, and all of them were anonymized. Written informed consent was required for the samples collected in Catalonia and were also anonymized; then, DNA extracts were submitted to the laboratory in Santiago de Compostela were the genotyping was carried out. In addition, the study was approved by the Ethical committee of the University of Santiago de Compostela. The study conforms to the Spanish Law for Biomedical Research (Law 14/2007- 3 of July).”

### Genotyping protocols and nomenclature

All the samples from Galicia, Cantabria, and Catalonia were sequenced for the HVS-I region (*N* = 518). Those samples belonging to R0 or with a dubious adscription to other non-R0 haplogroups (*N* = 293; ∼57%) were further genotyped for a set of 71 coding region SNPs mainly defining different branches within R0 (more information below). A total of 283 samples (∼55%) were finally classified as belonging to some R0 sub-branch.

The protocol for PCR amplification and automatic minisequencing is fully described in Text S1. Protocols for automatic sequencing of control region mtDNAs and complete genome sequencing are also shown in Text S1.

MtDNA variation is referred to the revised Cambridge Reference Sequence [20]. Haplogroup nomenclature is based on previous studies [1], [2], [3], [4], [5], [6], [7], [13], [15], [21], [22], [23]. As introduced above, an important number of conflicts have arisen among past studies, most of them because of the neglect of already existing nomenclature, or the delay of updating results from information available in the literature, or simply because overlapping of different publications. In order to reconcile the nomenclature conflicts between different studies, we have followed a chronological criterion when possible but only in case this nomenclature was harmonious with the almost worldwide accepted nomenclature rules and phylogenetic features [11].

### Monitoring genotyping errors

We have used the mtDNA tree as a reference to avoid as much as possible artefactual profiles and documentation errors in mtDNA sequences and in SNP genotypes [24], [25], [26], [27], [28], [29]. When detecting some unexpected SNP pattern, we confirmed the genotypes by repeating the SNP genotyping using single-plex minisequencing and automatic sequencing, as performed in Álvarez-Iglesias et al. [9].

### Genetic diversity estimates and analysis of geographical patterns

DnaSP 4.10.3 software [30] was used for the computation of different diversity indices, including haplotype and nucleotide diversities and mean number of pairwise differences [31], [32], [33]. Departure from normal distribution of pairwise differences was checked using the Harpending's r (raggedness) index [34]. Selective neutrality was tested using the Tajima [35] and Fu and Li tests [36].

The geographical representations of haplogroup frequencies were obtained using Surfer 8.0 (http://www.goldensoftware.com). The data used was collected from previous studies [3], [4], [6], [14] and the present one, representing a total of 24 different population samples. We used the inverse-squared distance method. Haplogroup frequencies are presented in a regular grid covering part of Eurasia (including Europe), Middle East and the Arabian Peninsula. Only data points within the same landmass, either island or continent, were considered for interpolation. In addition, we carried out analysis of spatial autocorrelation using the Spatial Autocorrelation Analysis Program (SAAP; http://www.exetersoftware.com/cat/saap.html) in order to detect and evaluate statistically signals of gradients (clines), gradients irradiating from the center of a particular area (depressions) or isolation by distance models; see for instance Barbujani [37].

### Phylogeographic analysis

R0 and its different sub-lineages are the main focus of the present article; however, there are only few studies focusing on the internal variability of R0 suitable for population comparisons [1], [3], [4], [14], [38], [39]. In addition, different haplotype searches were carried out using literature mtDNA datasets, most of them containing just HVS-I data; thus, in the literature there are more than 30,000 West Eurasian mtDNA profiles available that can be used for phylogeographic purposes.

Estimation of the time to the most recent common ancestor of each cluster and SDs were carried out according to Saillard et al. [40] and employing an evolutionary rate estimate which is intermediate between the one provided in [41] and [42], according to the procedures followed in [43]. Thus, the calibration corresponds now to 4,610 years per substitutions considering all the substitutions in the entire coding-region.

## Results

### The rationale for SNP selection and the R0 phylogeny

R0 differs from R* by lacking A73G and G11719A. R0 contains haplogroup HV which likewise embraces the most common haplogroup in Europe, H, but also haplogroup HV0a (where haplogroup V is nested) and some other minor branches such as HV1 and HV2. Within haplogroup H, there are at least 25 sub-haplogroups; many of them can be further sub-divided into minor branches.

MtDNA coding region SNP genotyping has been designed here with the aim of covering as much as possible the R0 phylogeny; given priority to those SNPs representing the most frequent sub-lineages, and also those characterizing branches that do not have any known diagnostic polymorphism in the control region. SNP selection in the present study considers the full set of SNPs reported in Brandstätter et al. [6] (with the exception of SNP A14552G which is replace here by C3936T; both leading to haplogroup H12) plus a selection of additional variants that define further sub-branches of R0 within Europe; see e.g. [4]. In addition, the analysis of the literature and complete genomes sequences available in GenBank has allowed us to infer new minor sub-lineages of R0 (see e.g. Text S2 and Table S1).

**Figure 1:**
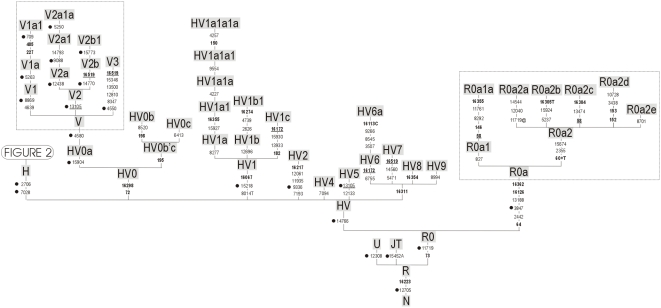
Phylogeny of haplogroup R0. An expanded view of the haplogroup H phylogeny is shown in Figure 2. Underlined positions signal parallel mutations, while @ indicates a back mutation. In bold are the control region variants, whereas dots indicate the SNPs selected and genotyped in the present study.

**Figure 2:**
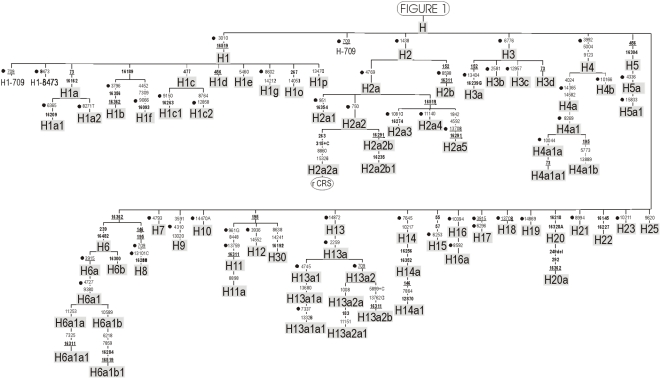
Phylogeny of haplogroup H. See legend of Figure 1 for more details.

When selecting mtDNA SNPs, it called our attention the many inconsistencies existing in the nomenclature of haplogroup H and its sub-lineages. One of the aims of the present study was therefore to resolve these nomenclature conflicts in order to ease inter-population genetic studies. These problems and the rationale to determine new sub-branches of R0 are shown in Text S2 and Table S1. The updated classification tree of haplogroup R0 and its sub-clades is shown in Figure 1 and Figure 2. These figures also indicate the SNPs selected and genotyped in the present study. We also incorporated in the minisequencing assays various diagnostic sites for haplogroups HV1 and HV2 (sister clades of H and HV0), and other polymorphisms covering several major branches of haplogroup R, namely, haplogroup U (A12308G) and JT (C15452A). The transition C12705T defining macro-haplogroup N was also added.

The advantages of using a minisequencing multiplex genotyping procedure *versus* other mtDNA SNP genotyping methods are reported and explained in Text S3. Some phylogenetic inconsistencies have been observed in our data, but all of them were confirmed by sequencing (see M&M); the most relevant ones are also described in Supplementary Data S2.

### Global mtDNA patterns in North Iberia

The three North Iberian samples analyzed in the present study show a typical West European mtDNA haplogroup composition (Table S2). Some haplogroups show slight differences in frequency. For example, while haplogroup H sums ∼39% of the total mtDNAs in Catalonia and Cantabria, it makes-up ∼44% of the mtDNA pool in Galicia. Haplogroup V reaches its highest frequency in Cantabria, ∼9%, and decrease substantially in Galicia (∼4%), and in Catalonia (∼3%). These differences in frequencies are not statistically significant (under a Chi-square test) but the patterns observed are in agreement with previous findings [5], [16].

All the HVS-I profiles obtained were searched among datasets compiled from the literature (more than 83.000 profiles) but only considering the common sequence range from position 16090 to 16365. A total of ∼5%, ∼10%, and ∼14% of the mtDNAs from Cantabria, Galicia and Catalonia, respectively, were still not observed in the literature. Catalonia shows the highest levels of sequence diversity, followed by Galicia and Cantabria (see also below and Table 1 for variability within haplogroup H). As expected, the most common haplotype was the one that matches the rCRS sequence, being very common in Galicia (∼20%; range 16090–16365), but decreasing in frequency towards Cantabria (∼13%) and Catalonia (∼12%).

**Table 1:** Summary statistics of HVS-I sequences in the North Iberian populations analyzed in the present article and other European populations.

HG	Population	*N*	*k*	*S*	*N_mut_*	*H*±SE	π±SE	*M*	*V*_O_(*M*)	*r*	*D*	*FL*
**All the sample**												
	Galicia*1	282	150 (0.53)	93	102	0.952±0.010	0.0138±0.001	3.76	5.06	0.012	−2.328**	−4.727**
	Catalonia*1	101	79 (0.78)	71	73	0.984±0.007	0.0166±0.001	4.59	6.29	0.014	−2.187**	−3.557**
	Cantabria*1	135	61 (0.45)	60	62	0.971±0.007	0.0135±0.001	3.72	3.85	0.018	−2.099*	−2.596*
**HG-H**												
	Galicia*1	124	51 (0.41)	49	50	0.800±0.038	0.006±0.001	1.73	2.08	0.035	−2.528***	−4.447**
	Catalonia*1	44	30 (0.68)	33	33	0.937±0.030	0.009±0.001	2.48	1.93	0.043	−2.300**	−3.836**
	Cantabria*1	52	26 (0.50)	25	26	0.875±0.042	0.006±0.001	1.78	1.33	0.067	−2.251**	−2.480**
	Volga-Ural*2	50	18 (0.36)	17	18	0.819±0.049	0.006±0.001	1.61	1.39	0.050	−1.884*	−1.966
	Finland*2	31	16 (0.52)	15	16	0.908±0.035	0.009±0.001	2.42	1.53	0.092	−1.338	−1.083
	Estonia*2	50	31 (0.62)	30	31	0.936±0.026	0.009±0.001	2.54	2.31	0.035	−2.114*	−3.113*
	Slovakia*2	50	30 (0.60)	31	30	0.939±0.027	0.009±0.001	2.49	2.23	0.045	−2.090*	−2.455*
	France*2	50	19 (0.38)	17	19	0.762±0.063	0.005±0.001	1.31	1.33	0.097	−2.187**	−2.569*
	Balkans*2	50	31 (0.62)	30	31	0.953±0.018	0.009±0.001	2.52	1.77	0.053	−2.120*	−2.852*
	Turkey*2	50	31 (0.62)	27	31	0.914±0.032	0.008±0.001	2.13	1.59	0.055	−2.311**	−3.113*
	Near East*2	50	36 (0.72)	30	36	0.943±0-023	0.009±0.001	2.56	2.08	0.040	−2.301**	−4.097**
	Asia*2	48	29 (0.60)	26	29	0.947±0.019	0.010±0.001	2.89	2.37	0.029	−1.962*	−2.261
	Eastern Slavs^2^	50	30 (0.60)	31	30	0.944±0.023	0.009±0.001	2.35	1.67	0.057	−2.162*	−3.280*
	Arabian Peninsula*3	52	29 (0.56)	30	30	0.947±0.017	0.008±0.001	2.32	1.34	0.074	−2.153*	−3.050*
	Armenia*3	54	27 (0.50)	33	33	0.914±0.031	0.009±0.001	2.53	2.35	0.030	−2.158*	−1.685
	Daghestan*3	60	26 (0.43)	33	33	0.859±0.042	0.008±0.001	2.17	2.28	0.023	−2.268**	−2.323
	Georgia*3	30	15 (0.50)	16	16	0.874±0.050	0.008±0.001	2.11	2.12	0.031	−1.617	−0.682
	Jordan*3	33	18 (0.55)	25	25	0.847±0.062	0.008±0.001	2.24	2.30	0.024	−2.227**	−2.586*
	Karatchaians-Balkanians*3	50	21 (0.42)	23	23	0.943±0.017	0.012±0.001	3.23	2.00	0.059	−1.202	0.411
	Lebanon*3	34	20 (0.59)	23	23	0.907±0.041	0.008±0.001	2.09	1.88	0.061	−2.171*	−3.548**
	Northwest Caucasus*3	69	35 (0.51)	38	38	0.895±0.034	0.009±0.001	2.42	2.70	0.026	−2.256**	−2.953*
	Ossetia*3	45	22 (0.49)	26	27	0.883±0.002	0.009±0.001	2.58	2.84	0.029	−1.950*	−2.445
	Syria*3	28	19 (0.68)	23	23	0.966±0.019	0.009±0.001	2.38	1.38	0.098	−2.139*	−2.667*
	Turkey*3	90	46 (0.51)	44	46	0.898±0.029	0.008±0.001	2.24	2.10	0.037	−2.408**	−2.957*
	Austria*4	964	116 (0.12)	75	81	0.683±0.017	0.005±0.001	1.15	1.07	0.041	−2.468***	−5.322
	Germany*4	28	20 (0.71)	20	20	0.952±0.030	0.010±0.001	2.73	1.88	0.042	−1.657	−1.116
	Hungary*4	55	15 (0.27)	22	22	0.677±0.070	0.006±0.001	1.64	2.61	0.073	−2.059*	−2.160
	Macedonia*4	88	30 (0.34)	28	29	0.892±0.025	0.007±0.001	2.01	1.84	0.058	−2.000*	−1.707
	Romania*4	100	29 (0.29)	29	29	0.917±0.017	0.009±0.001	2.48	2.04	0.034	−1.690	−2.160

*N* = sample size, *k* = number of different haplotypes (divided by *N* in brackets).

*S* = Number of polymorphic (segregating) sites.

*N_mut_* = total number of mutations.

*H* = haplotype diversity and standard error.

π = nucleotide diversity and standard error.

*M* = average number of nucleotide differences.

*V*_O_(*M*) = observed variance of *M*.

*r* = Harpending's (raggedness) index.

*D* = Tajima's test of selective neutrality.

*FL* = Fu and Li's D* statistics.

Statistical significance: *, *P*-value<0.05.

** *P*-value<0.02.

*1 Present study.

*2 Loogväli et al. [3].

*3 Roostalu et al. [4].

*4 Brandstätter et al. [15].

A small percentage of the total mtDNAs analyzed belonged to non-Eurasian lineages. Thus, several sub-Saharan mtDNA profiles were detected in Galicia (∼2.5%) and Catalonia (∼3%); none in Cantabria. Curiously, six out of the ten sub-Saharan haplotypes observed belong to haplogroup L1b; this clade originated in western Africa but it was also carried to America during the period of the Atlantic slave trade [44], [45], [46]. For instance, the L1b1 profile found in Galicia, T16126C C16187T T16189C C16223T C16264T C16270T C16278T A16293G T16311C (note also the presence in Galicia of another derivative haplotype with A16317G on top), is found in many sub-Saharan regions [47], [48], but also in America [49], [50], [51]. The rare haplotype, C16169T C16193T T16195C C16223T T16243C C16261T, observed in Galicia belongs to the uncommon sub-Saharan haplogroup L3x2 typically found in Ethiopia and Yemen [52]; peculiarly, this haplotype was also detected in an independent sample collected from the same region some years ago [16], [17].

Some typical Native American profiles were also observed in the Catalonian sample. For instance, the haplogroup D1 profile T16189C C16223T T16325C T16362C (excluding the ‘speedy’ transversion A16183C) is commonly found in South America [53], [54], or the A2 haplotype C16111T T16189C C16223T C16290T G16319A T16362C (excluding highly mutable transition T16519C), which is also common in all around America [49], [50].

In Catalonia we have also observed one rare East Asian profile, C16104T C16111T T16140C A16162G 16169+C A16182C A16183C T16189C C16228T C16234T T16243C, belonging to B5b. Members of this haplogroup appear frequently in Japan, Taiwan, Korea, etc. [9], [55].

The presence at low frequency of non-western European lineages in Catalonia could be explained by recent gene flow because it is well-known that this region has received important flow of immigrants in the last decades; more than Galicia and Cantabria.

### Diversity patterns of R0 in North Iberia

Several diversity indices were computed for the three North Iberian samples analyzed in the present study (Table 1). Overall, the Catalonian sample shows the highest values of sequence and nucleotide diversity (however with overlapping ranges considering a confidence interval of two standard errors) and also for the average number of nucleotide differences. The Cantabrian region shows the lowest values again for the three mentioned indices.

The patterns of variability within haplogroup H are quite different around Europe and Middle East. For instance, Galicia shows one of the lowest sequence diversity values within Eurasia (Table 1), in agreement with a previous independent study from the region [16]); and it is also among the regions with lowest values of nucleotide diversities (together with Cantabria).

Both the Tajima's *D* and the Fu and Li's tests show significantly negative values in almost all the populations (Table 1), suggesting that all of them have passed through population expansions. The mismatch distributions (data not shown) also support this hypothesis as well as the raggedness *r* index (Table 1) indicating that the mismatch distributions are unimodal and then compatible with population expansion.

### Phylogeographical patterns of R0 sub-lineages

Using the SNP genotyping strategy described above, less than 10% of the lineages within haplogroup H could not be allocated to some of the already known H sub-branches (Table S3).

The distribution of haplogroup frequencies along the North Iberian fringe shows patterns moderately stratified.

On average, ∼42% of the mtDNAs in the total sample belongs to haplogroup H; the Galician sample reaches the highest frequency (∼44%), and it is slightly lower (∼39%) in Cantabria and Catalonia.

H* represents 15% and 10% of the total haplogroup H lineages in Catalonia and Galicia, respectively, but only 4% in Cantabria. H1a (without counting H1a1 and H1a2) represents 8% of the haplogroup H mtDNAs in Cantabria, but it makes-up only 2% in Galicia and 0% in Catalonia. Again with respect to haplogroup H, H1 is more frequent also in Cantabria (46%) than in Galicia (38%) and Catalonia (36%); whereas haplogroup V has a clear peak in Cantabria, ∼16% of the total R0 haplotypes (but only ∼9% in Galicia and ∼6% in Catalonia).

**Figure 3:**
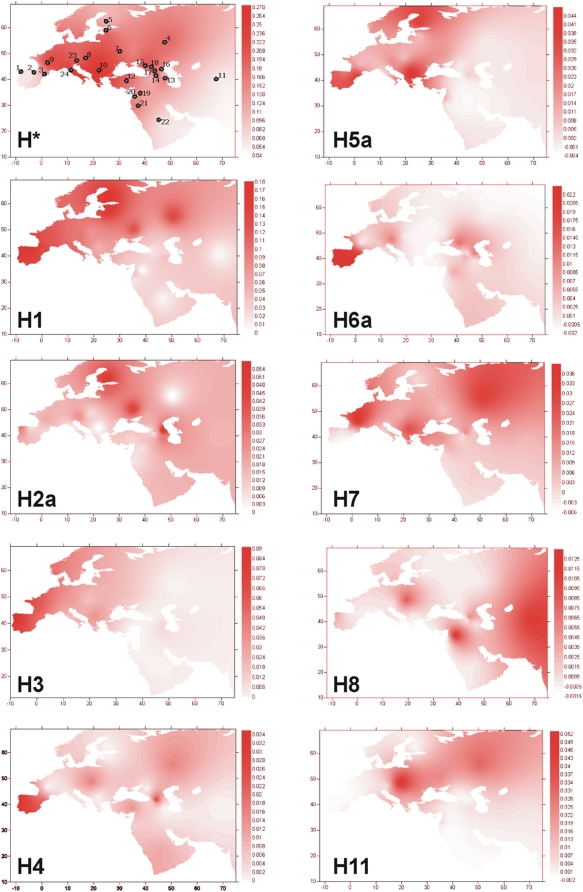
Geographic maps of haplogroup frequencies for haplogroups H*, H1, H2a, H3, H4, H5a, H6a, H7, H8, H11. Dots in the map of H* indicate the location of the populations used. Codes for populations are: [1] Galicia, [2] Cantabria, [3] Catalonia (present study); [4] Volga-Ural, [5] Finland, [6] Estonia, [7] Eastern Slavs, [8] Slovakia, [9] France, [10] Balkans, [11] Central Asia [3]; [12] Turkey, [13] Armenia, [14] Georgia, [15] Northwest Caucasus, [16] Daghestan, [17] Ossetia, [18] Karatchians-Balkarians, [19] Syria, [20] Lebanon, [21] Jordan, [22] Arabian Peninsula [4]; [23] Austria [6]; [24] Tuscani [14].

The maps of Figure 3 show the spatial frequency distribution of different sub-lineages of haplogroup H. Some clades get the highest frequency in Iberia, such as H1, H3, and H5a or are only observed in this region (H4); while others are virtually absent in Iberia but are significantly more prevalent in Central Europe (e.g. H11).

In addition, haplogroups H1, H3, and H5a display clinal patterns as determined by their spatial correlograms (Figure S1). The frequency of these three haplogroups has a peak in the Franco-Cantabrian refuge area and declines towards East Europe.

### The autochthonous nature of the H2a5 clade in the Basque Country

It was first notice in a study by Pereira et al. [19] by way of sequencing several small coding region mtDNA segments, the presence of the coding region variant C4952T in ∼6% of their samples from the Basque Country.

A scrutiny of more than 5,500 coding region segments (most of them available in GenBank and some only in the literature) and in Google searches (*sensu* [56], [57]) revealed that this variant was only reported twice, curiously in two medical studies [58], [59] where no detailed information on the geographical origin of the carriers was provided. Therefore, the multiple occurrence of this transition in the Basque Country could point to a diagnostic site for an autochthonous lineage in this region. These features lead us to further investigate these mtDNAs by way of complete genome sequencing the eight available Basque samples carrying transition C4592T.

**Figure 4:**
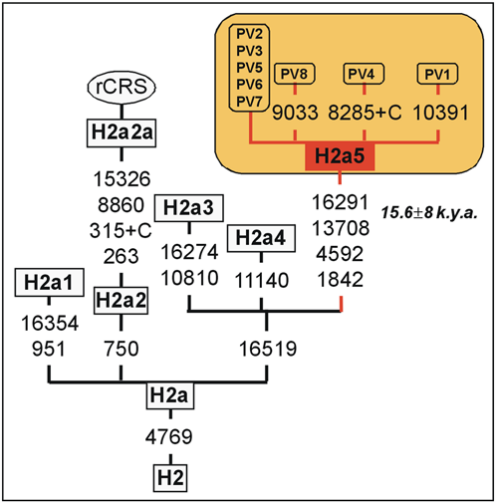
Phylogeny of haplogroup H2a5.

This analysis revealed a new sub-clade of haplogroup H, baptized here as H2a5. All these sequences share the following diagnostic variants: A1842G C4592T G13708A C16291T T16519C (Figure 4). Six out of the eight complete genomes are identical while the other two show one private variant each. The coalescence age for this sub-lineage is 15.7±9 kya.

## Discussion

Analysis of mtDNA variation based exclusively on few RFLP markers and/or the HVS-I region have lead in the past to simplistic perceptions of Europe as a uniform population. The results presented in previous studies [1], [3], [4], [6], [15], [60] and those shown here, demonstrate that population stratification in European population can only be revealed when using higher phylogenetic resolution. Analysis of complete genomes ideally provides the maximum level of resolution; however, genotyping complete mtDNA molecules demands great economical and personal effort in large-scale population projects. Complete genome sequencing is generally carried out when the analysis focuses on a particular group of mtDNAs presenting some interesting phylogenetic or population feature [1], [3], such as performed here for the analysis of members belonging to haplogroup H2a5. At a population level, the coding region genotyping strategy presented here represents a way to overcome the drawback of whole genome genotyping and allow at the same time obtaining high resolution information from the mtDNA genome.

A total of 518 samples from three main locations in North Iberia were sequenced for the HVS-I segment. About 55% of them could be ascribed to R0. All these samples were further screened for a set of 71 coding region SNPs in order to sub-classify them into different R0 sub-clades. As indicated by the various diversity indices computed, Galicia and Cantabria show low diversity values, especially for the overall haplogroup H. The present study also revealed moderate levels of stratification in North Iberia, which could be relevant in other fields of research, such as in forensic casework [61] or in medical studies, where population sub-structure could explain most of the false positives of association in case-control studies [62].

When compared to other European and Middle East populations, we observed geographical patterns for H1, H3 and H5a that are statistically clinal, with frequency peaks in the Franco-Cantabrian region decreasing towards East Europe. This is compatible with a process of demographic repopulation of Europe after the LGM period centered in this climatic and geographic refuge, as it was previously demonstrated by Torroni et al. [5] and Achilli et al. [1].

We have also described a new minor autochthonous clade in Basques, H2a5. This lineage has been dated in 15.6±8 kya; this age fits also with the period of population expansion that followed the LGM (although with a large standard error). However, this branch was exclusively found in the Basque country at a significant frequency (∼6%). The absence of this clade in other parts of Europe could be due to the limited sample size still available in the literature; however, we can speculate with the fact that all the evidences taken together resemble the findings of Torroni et al. [5] and Achilli et al. [1] regarding the ‘imprint’ of post-LGM human population re-expansions centered in the Franco-Cantabrian refuge on the mtDNA variability.

## Supporting Information

Text S1 Genotyping protocols.
(0.27 MB DOC)

Text S2 Reconciliation of the nomenclature conflicts in haplogroup R0.
(0.14 MB DOC)

Text S3 Note about the advantages of using minisequencing high throughput SNP genotyping and report of the phylogenetic inconsistencies observed in the data from North Iberia.
(0.04 MB DOC)

Table S1 Compendium of the problems related to the nomenclature of the R0 phylogeny and update of the nomenclature.
(0.05 MB XLS)

Table S2 HVS-I and coding region SNP variation for the Iberian samples analyzed in the present study.
(0.71 MB XLS)

Table S3 Comparative population frequencies of different haplogroup H (sub)lineages. In bold we collapse frequencies into higher hierarchical phylogenetic clades as a function of the SNPs genotyped in the referred studies, such that only these ‘bolded’ categories are fully comparable between the different studies considered. This is because haplogroup categories are not fully comparable among populations when the samples have undetermined (nd) SNPs; for instance, H* embraces different lineages in our study because we genotyped to a higher level of resolution than previous attempts (where different lineages were already collapsed into H*). For nomenclature we follow the scheme of Figure 1 and Figure 2.
(0.08 MB XLS)

Figure S1 Autocorrelograms for the most frequent R0 sub-clades observed in North Iberia.
(0.12 MB PPT)
