# Correction: New Population and Phylogenetic Features of the Internal Variation within Mitochondrial DNA Macro-Haplogroup R0

**DOI:** 10.1371/annotation/25b9ed74-e963-496e-84bb-526bd6867221

**Published:** 2010-04-27

**Authors:** Vanesa Álvarez-Iglesias, Ana Mosquera-Miguel, Maria Cerezo, Beatriz Quintáns, Maria Teresa Zarrabeitia, Ivon Cuscó, Maria Victoria Lareu, Óscar García, Luis Pérez-Jurado, Ángel Carracedo, Antonio Salas

There was an error in the computation of the age of haplogroup H2a5. It is about 1285 ± 161 years old according to the calibration rate used in the manuscript. Its recent age, and assuming a local episode of isolation, might easily explain its confined location to the Basque Country. This sub-clade has therefore nothing to do with the post-glacial expansions affecting other lineages e.g. haplogroup V. We would like to thank Vicente Cabrera for kindly let us know about this error. 

