# Supplementary material for: New Population and Phylogenetic Features of the Internal Variation within Mitochondrial DNA Macro-Haplogroup R0
Source: PLoS One. 2009 Apr 2;4(4):e5112. doi: 10.1371/journal.pone.0005112 (PMC2660437; doi:10.1371/journal.pone.0005112)
Supplement: Text S1 — Genotyping protocols. (0.27 MB DOC) [file pone.0005112.s001.doc]

**Text S1.**

*Amplification and minisequencing protocols*

Primer3 software (http://frodo.wi.mit.edu/cgi-bin/primer3) was used for designing the primers both for PCR amplification and minisequencing reaction. All of them have an annealing temperature around 60º C. The sequence databases at the National Centre for Biotechnology Information (NCBI; http://www.ncbi.nlm.nih.gov) were interrogated using BLAST in order to test the primers against possible repetitive sequences and sequence homologies in the nuclear genome. Each primer pair for PCR amplification and each single base extension primer were selected independently and AutoDimer (http://www.cstl.nist.gov/biotech/strbase/AutoDimerHomepage/AutoDimerProgramHomepage.htm) was used to test for potential hairpin structures and primer-dimer problems.

The SNPs were amplified in three independent PCR multiplex reactions. The size of the amplicons ranges from 66 and 195 bp. Some SNPs are located in the same amplicon, reducing substantially the number of amplicons needed in the whole multiplex design. We performed each multiplex reaction using 5 ng of DNA template and PCR master mix of QIAGEN Kit Multiplex PCR (Qiagen, Dusseldorf, Germany), amplification primers and their final concentrations are in Table 1 to Table 3 below. Amplification was carried out in a 9700 Thermocycler (Applied Biosystems, Foster City, CA, USA). After a 95º C pre-incubation step for 15 minutes, PCR was performed in a total of 30 cycles using the following conditions: 94º C denaturation for 30 seconds, annealing at 58º C for 90 seconds and extension at 72º C during 90 seconds, followed by a 15 minutes of final extension at 72º C and 4º C until removed from thermocycler.

Previous to minisequening reaction, PCR products were treated with ExoSAP-IT (Amershan Biosciences, Uppsala, Sweden) to remove excess of primers and un-incorporated dNTPs: 1 μl of PCR product was incubated together with 0.5 μl of ExoSAP-IT for 15 minutes at 37º C followed by 15 minutes at 85º C for enzyme inactivation. Minisequencing reaction was performed using SNaPshot^TM^ Kit (AB). Minisequencing primers range in size from 21 to 86 bps, length primers were modified by the addition of non-homologous tails, poly(dGACT) added at the 5’- end (Table 4 to Table 6). The minisiquencing reaction was carried out in a total volume of 10 μl comprising 3 μl of the SNaPshot^TM^ Kit (AB), 1.5 μl PCR product, 1 μl of extension primers mix (final concentrations are between 0.06 and 0.6 µM), and water-up to 10 μl. The reaction was performed in a 9700 Thermocycler (AB) following the recommendations of the manufacturer: 25 cycles of denaturation at 96º C for 10 seconds, annealing at 50º C for 5 seconds and extension at 60º C during 30 seconds. Un-incorporated ddNTPs were eliminated using SAP (Amershan Biosciences). The final volume (10 μl) was treated with 1 μl of SAP for 60 minutes at 37º C followed by 15 minutes at 85º C for enzyme inactivation.

The minisiquencing products (2 μl) were mixed with 9.5 μl of HiDi^TM^ formamide (AB) and 0.3 μl of GeneScan –120 LIZ (AB) and capillary electrophoresis was undertaken on an ABI PRISM 3130xl Genetic Analyzer (AB) .Resulting data were analyzed using GeneMapper^TM^ 3.7 Software (AB).

Figure 1 below shows an example of a minisequencing electropherogram.

*Automatic sequencing of control region*

All the samples from Galicia, Cantabria, and Catalonia (*N*=518) were sequenced for the HVS-I. PCR amplification was carried out in a 9700 Thermocycler (AB). The temperature profile for 32 cycles of amplification was 95º C for 10 seconds, 60º C for 30 seconds, and 72º C for 30 seconds. Sequencing primers were previously described by (Wilson et al. 1995). PCR product purification and sequencing were performed as in Salas et al. (1998). For those sequences containing a continuous homopolymeric cytosine stretch from positions 16184 to 16193 (usually related to length heteroplasmy), additional amplification and sequencing were performed in order to fully cover this region.

*Genotyping transition C4592T*

Using the same protocol described above (for primers see the corresponding amplicon 4550-4580 described in Table 1), transition C4592T was sequenced in all the haplogroup H2 mtDNAs observed in our samples from Galicia, Cantabria, and Catalonia. None of them carried this transition.

*Complete genome genotyping*

The eight samples collected in the Basque Country belonging to H2a5 were sequenced for the complete genome. Primers used for PCR amplification and sequencing were those reported in (Torroni et al. 2001). PCR was performed in 10µl of reaction mix, containing 4 µl of PCR Master Mix (Qiagen), 0.5 µl 1µM of each primer, 1 µl sample template and 4 µl of water. This PCR was carried out in a 9700 Thermocycler (AB) with one cycle of 95º C for 15 minutes and then 35 cycles of 94º C for 30 seconds, 58º C for 90 seconds and 72º C for 90 seconds with a full extension cycle of 72º C for 10 minutes. The PCR product was checked in agarose gels and purified using QIAquick^R^ Gel Extraction Kit (Qiagen).

Sequencing reaction was performed in 11.5 µl of reaction mixture, containing 2.5µl of sequencing buffer (5X), 0.5 µl of BigDye Terminator v3.1 Cycle Sequencing Kit (Applied Biosystems), 1 µl of the corresponding primer (final concentration was 1 µM), 3 µl of the purified PCR product and water up to 11.5 µl. Sequencing reaction was carried out in a 9700 Thermocycler (AB) with one cycle of 96º C for 3 minutes and then 25 cycles of 96º C for 30 seconds, 50º C for 15 seconds and 60º C for 4 minutes or was carried out in a 9800 Fast Termal Cycler (Applied Biosystems) with one cycle of 96º C for 1 minute then 25 cycles of 96º C for 10 seconds, 50º C for 5 seconds and 60º C for 1 minute. To obtain ‘clean’ electropherograms, the sequencing product was doubled purified, first using Montage^TM^ SEQ_96_ Sequencing Reaction Cleanup Kit (Millipore) according to manufacturer protocols, followed by purification with Sephadex^TM^ G-10 (Amershan Biosciences, Uppsala, Sweden), the latter also according to manufacturer protocol. MtDNA automatic sequencing was carried out in a capillary electrophoresis ABI3730 (Applied Biosystems).

*References*

Salas A, Comas D, Lareu MV, Bertranpetit J, Carracedo Á (1998) mtDNA analysis of the Galician population: a genetic edge of European variation. Eur J Hum Genet 6(4): 365-375.

Torroni A, Rengo C, Guida V, Cruciani F, Sellitto D et al. (2001) Do the four clades of the mtDNA haplogroup L2 evolve at different rates? Am J Hum Genet 69(6): 1348-1356.

Wilson MR, DiZinno JA, Polanskey D, Replogle J, Budowle B (1995) Validation of mitochondrial DNA sequencing for forensic casework analysis. Int J Legal Med 108(2): 68-74.

**Table 1.** Amplification primers for multiplex 1. In the SNP column indicates the SNP genotyped in each amplicon. F.C. = final concentration

| **SNP** | **Primer forward and reverse** | **Size (bp)** | **F.C. (µM)** |
| --- | --- | --- | --- |
| 709/750 | GGCTCACATCACCCCATAAA  CGTTTTGAGCTGCATTGCT | 161 | 0.2 |
| 2581/2706 | GCCTGCCCAGTGACACAT  GCTCCATAGGGTCTTCTCGT | 195 | 0.2 |
| 3010 | CAATAACTTGACCAACGGAACA  CGGTCTGAACTCAGATCACGTA | 179 | 0.4 |
| 3796/3847 | TCAACATTACTAATAAGTGGCTCCTTT  GGTTCGGTTGGTCTCTGCTA | 135 | 0.2 |
| 4550/4580 | CAACCCGTCATCTACTCTACCAT  CTTCTGTGGAACGAGGGTTTATT | 148 | 0.2 |
| 6253/6296/6365 | TGACTCTTACCTCCCTCTCTCC  GATGAAATTGATGGCCCCTA | 189 | 0.2 |
| 6776 | GCTTCCTAGGGTTTATCGTGTG  GAGTGTGGCGAGTCAGCTAAA | 140 | 0.4 |
| 7337 | GGCTCATTCATTTCTCTAACAGC  TCCAGGTTTATGGAGGGTTC | 110 | 0.4 |
| 10810 | GCTAAAACTAATCGTCCCAACA  AATTAGGCTGTGGGTGGTTG | 97 | 0.2 |
| 12858/12957 | CAACACAGCAGCCATTCAAG  GAGGCCTAGTAGTGGGGTGA | 157 | 0.2 |
| 13708/13759 | AACGAAAATAACCCCACCCTA  GTTGTTTGGAAGGGGGATG | 113 | 0.2 |
| 14365/14470 | CCACCCCATCATACTCTTTCA  TAGGGGGAATGATGGTTGTC | 159 | 0.3 |
| 14766/14770A | TCAACTACAAGAACACCAATGACC  GGAGGTCGATGATGAGTGG | 82 | 0.2 |
| 15218 | ACTATCCGCCATCCCATACA  GGGCAAGATGAAGTGAAAGG | 110 | 0.4 |

**Table 2.** Amplification primers for multiplex 2. In the SNP column indicates the SNP genotyped in each amplicon. F.C. = final concentration

| **SNP** | **Primer Forward and Reverse** | **Size (bp)** | **F.C. (μM)** |
| --- | --- | --- | --- |
| 951/961G | TCACACGATTAACCCAAGTCA  ACTCAGGTGAGTTTTAGCTTTATTG | 87 | 0.2 |
| 3915/3936/3992 | TAGCAGAGACCAACCGAACC  GAAGATTGTAGTGGTGAGGGTGT | 158 | 0.6 |
| 4310/4336 | AGCATTCCCCCTCAAACCTA  TTTTGGATTCTCAGGGATGG | 127 | 0.4 |
| 4727/4745/4769/4793 | TCCTTCTAATAGCTATCCTCTTCAACA  TGGGTAACCTCTGGGACTCA | 154 | 0.4 |
| 7028 | CACCGTAGGTGGCCTGACTGGC  GTGTAGCCTGAGAATAGGGG | 168 | 0.4 |
| 7645 | ACATGCAGCGCAAGTAGGTC  AAAATGATTATGAGGGCGTGA | 90 | 0.2 |
| 8269/8271T | TAGGGCCCGTATTTACCCTAT  AAGAGGTGTTGGTTCTCTTAATCTTT | 110 | 0.2 |
| 8473/8592/8598/8602 | CCCAACTAAAAATATTAAACACAAACT  GGAGGTGGGGATCAATAGAG | 193 | 0.2 |
| 9066/9088/9150 | CCTACTCATGCACCTAATTGGA  GGCTTACTAGAAGTGTGAAAACGTA | 155 | 0.3 |
| 10044 | CCGTTAACTTCCAATTAACTAGTTTTG  AAGGCTAGGAGGGTGTTGATT | 91 | 0.6 |
| 10394 | CCATGAGCCCTACAAACAACT  TGAGTCGAAATCATTCGTTTTG | 159 | 0.3 |
| 13404 | TATGTGCTCCGGGTCCATC  TGGTGAGGGAGGTTGAAGTG | 104 | 0.2 |

**Table 3.** Amplification primers for multiplex 31. In the SNP column indicates the SNP genotyped in each amplicon. F.C. = final concentration

| **SNP** | **Primer Forward and Reverse** | **Size(bp)** | **F.C. (μM)** |
| --- | --- | --- | --- |
| 1438 | AACTTAAGGGTCGAAGGTGGA  AGGGCCCTGTTCAACTAAGC | 66 | 0.1 |
| 2259 | TCAAGCTCAACACCCACTACC  TGCGGAGGAGAATGTTTTCA | 131 | 0.2 |
| 5250/5263 | ATTCCATCCACCCTCCTCTC  GGTGGGGATGATGAGGCTAT | 111 | 0.6 |
| 8869 | GGACTCCTGCCTCACTCATTT  AAGTGGGCTAGGGCATTTTT | 128 | 0.4 |
| 8994 | AATGCCCTAGCCCACTTCTT  AGGTGGCCTGCAGTAATGTT | 140 | 0.1 |
| 9336 | GCCATGTGATTTCACTTCCA  GTGGCCTTGGTATGTGCTTT | 117 | 0.2 |
| 10166/10211 | ACTACCACAACTCAACGGCTACA  AGGGGTAAAAGGAGGGCAAT | 145 | 0.2 |
| 11140 | CATTCACAGCCACAGAACTAATCAT  GTTCTGGCTGGTTGCCTCAT | 99 | 0.2 |
| 11719 | CAGCCATTCTCATCCAAACC  GCGTTCGTAGTTTGAGTTTGC | 113 | 0.3 |
| 12308 | CTGCTAACTCATGCCCCCATG  ATTACTTTTATTTGGAGTTGCACCAAGATT | 106 | 0.3 |
| 12438 | CCACCCTAACCCTGACTTCC  GTGGATGCGACAATGGATTT | 106 | 0.1 |
| 12705 | TGTAGCATTGTTCGTTACATGG  AGTTGGAATAGGTTGTTAGCGG | 147 | 0.2 |
| 13101C/13105 | CAGCCCTACTCCACTCAAGC  TGGGCTATTTTCTGCTAGGG | 83 | 0.2 |
| 14869/14872 | CAACATCTCCGCATGATGAA  AGGCGTCTGGTGAGTAGTGC | 104 | 0.2 |
| 15452A | AGACGCCCTCGGCTTACTT  GTCGCCTAGGAGGTCTGGTG | 78 | 0.2 |
| 15773 | CCGCAGACCTCCTCATTCTA  CGGATGCTACTTGTCCAATG | 81 | 0.1 |
| 15833/15904 | CCCTTTTACCATCATTGGACA  AAAGGTTTTCATCTCCGGTTT | 162 | 0.2 |

**Table 4.** Minisequencing primers for multiplex 1. In the SNP column indicates the SNP genotyped in each amplicon. F.C. = final concentration

| **SNP** | **Minisequencing primer** | **Size (pb)** | **Base change** | **Chain** | **F.C. (µM)** |
| --- | --- | --- | --- | --- | --- |
| 709 | (gact)_3_TTACACATGCAAGCATCCCC | 32 | G-A | L | 0.2 |
| 750 | CTCTAAATCACCACGATCAAAAGG | 24 | A-G | L | 0.2 |
| 2581 | TGATTATGCTACCTTTGCACGGT | 23 | A-G | H | 0.2 |
| 2706 | (gact)_2_gAGGGTCTTCTCGTCTTGCTGTGT | 32 | A-G | H | 0.2 |
| 3010 | (gact)_3_gAACCTTTAATAGCGGCTGCACCAT | 37 | G-A | H | 0.2 |
| 3796 | (gact)_2_gaCTAATAAGTGGCTCCTTTAACCTCTCC | 37 | A-G | L | 0.1 |
| 3847 | (gact)_5_ATTACTCCTGCCATCATGACCC | 42 | T-C | L | 0.2 |
| 4550 | (gact)_13_gaGCGCTAAGCTCGCACTGATT | 74 | T-C | L | 0.3 |
| 4580 | (gact)_3_gaTTACCTGAGTAGGCCTAGAAATAAACAT | 42 | G-A | L | 0.2 |
| 6253 | (gact)_6_gaTGTTCCTGCTCCGGCCTCCACT | 48 | T-C | H | 0.1 |
| 6296 | (gact)_6_AACAGGTTGAACAGTCTACCCTCC | 48 | C-T | L | 0.2 |
| 6365 | (gact)_7_gaGATGGCCCCTAAGATAGAGGAGAC | 54 | T-C | H | 0.2 |
| 6776 | (gact)_6_acCGTGTGTCTACGTCTATTCCTACTGTAAATAT | 58 | T-C | H | 0.3 |
| 7337 | (gact)_10_TGATTTGAGAAGCCTTCGCTTC | 62 | G-A | L | 0.3 |
| 10810 | (gact)_5_gacCAACAATTATATTACTACCATTGACATGACT | 54 | T-C | L | 0.2 |
| 12858 | (gact)_8_gacGCAGCCATTCAAGCAATCCTATA | 58 | C-T | L | 0.3 |
| 12957 | (gact)_9_CAACAAATAGCCCTTCTAAACGCTAA | 62 | T-C | L | 0.3 |
| 13708 | (gact)_13_ gaCTACTAAACCCCATTAAAGGCCTG | 78 | G-A | L | 0.3 |
| 13759 | (gact)_10_TTCTCATTACTAACAACATTTCCCCC | 66 | G-A | L | 0.1 |
| 14365 | (gact)_11_ gaGTTAGCGATGGAGGTAGGATTGGT | 70 | C-T | H | 0.3 |
| 14470A | (gact)_13_gCCTCAATAGCCATCGCTGTAGTATA | 78 | T-A | L | 0.4 |
| 14770 | (gact)_11_GAATGAGTGGTTAATTAATTTTATTAGGGG | 74 | C-T | H | 0.3 |
| 14766 | (gact)_11_ gCAATGACCCCAATACGCAAAA | 66 | T-C | L | 0.3 |
| 15218 | (gact)_11_TCCTCAGATTCATTGAACTAGGTCTG | 70 | A-G | H | 0.3 |

**Table 5.** Minisequencing primers for multiplex 2. In the SNP column indicates the SNP genotyped in each amplicon. F.C. = final concentration

| **SNP** | **Minisequencing primer** | **Size (pb)** | **Base change** | **Chain** | **F.C. (µM)** |
| --- | --- | --- | --- | --- | --- |
| 951 | CTTTATTGGGGAGGGGGTGAT | 21 | G-A | H | 0.2 |
| 961G | (gact)_15_CGTAAAGAGTGTTTTAGATCACCCCC | 86 | T-G | L | 0.06 |
| 3915 | (gact)_2_GAAGCCTGAGACTAGTTCGGACTC | 32 | G-A | H | 0.2 |
| 3936 | (gact)_14_gTGCGGCGTATTCGATGTTGAA | 78 | C-T | H | 0.6 |
| 3992 | (gact)_3_gaCCCTATTCTTCATAGCCGAATACA | 38 | C-T | L | 0.3 |
| 4310 | TCTGATAAAAGAGTTACTTTGATAGAGTAAATAATAGG | 38 | A-G | L | 0.4 |
| 4336 | AGGGATGGGTTCGATTCTCAT | 21 | T-C | H | 0.2 |
| 4727 | (gact)_5_gaTACTCTCCGGACAATGAACCAT | 44 | A-G | L | 0.2 |
| 4745 | gactAATGAACCATAACCAATACTACCAATCA | 32 | A-G | L | 0.2 |
| 4769 | (gact)_4_gaACCAATCAATACTCATCATTAATAATCATAAT | 50 | A-G | L | 0.2 |
| 4793 | (gact)_5_gATAATCATAATAGCTATAGCAATAAAACTAGGAAT | 56 | A-G | L | 0.4 |
| 7028 | (gact)_4_gacTACACGACACGTACTACGTTGTAGC | 44 | C-T | L | 0.2 |
| 7645 | (gact)_6_GCTACTTCCCCTATCATAGGAGAGCT | 50 | T-C | L | 0.2 |
| 8269 | (gact)_8_gacTGAAATAGGGCCCGTATTTACCCTATA | 62 | G-A | L | 0.06 |
| 8271T | (gact)_15_GGGCCCGTATTTACCCTATAGC | 82 | A-T | L | 0.3 |
| 8473 | (gact)_6_gaAAAAATATTAAACACAAACTACCACCTACC | 56 | T-C | L | 0.2 |
| 8592 | (gact)_11_gacGCCTACCCGCCGCAGTACT | 66 | G-A | L | 0.1 |
| 8598 | (gact)_15_gacACCCGCCGCAGTACTGATCAT | 84 | T-C | L | 0.4 |
| 8602 | (gact)_13_ CGCCGCAGTACTGATCATTCTA | 74 | T-C | L | 0.4 |
| 9066 | (gact)_8_gaGTGTAGAGGGAAGGTTAATGGTTGATAT | 62 | A-G | H | 0.3 |
| 9088 | (gact)_10_AGTAGAATTAGAATTGTGAAGATGATAAGTGTAG | 74 | T-C | H | 0.2 |
| 9150 | (gact)_12_gCCTAGAAATCGCTGTCGCCTT | 70 | A-G | L | 0.2 |
| 10044 | (gact)_9_TTAAGGCGAAGTTTATTACTCTTTTTTGAA | 66 | A-G | H | 0.6 |
| 10394 | (gact)_9_gaCTGGCCTATGAGTGACTACAAAAAGGATTAGA | 70 | C-T | L | 0.3 |
| 13404 | (gact)_11_gaATCATCCACAACCTTAACAATGAACAAGATAT | 78 | T-C | L | 0.3 |

**Table 6.** Minisequencing primers for multiplex 3. In the SNP column indicates the SNP genotyped in each amplicon. F.C. = final concentration

| **SNP** | **Minisequencing primer** | **Size (pb)** | **Base change** | **Chain** | **F.C. (µM)** |
| --- | --- | --- | --- | --- | --- |
| 1438 | (gact)_10_gaGTCGAAGGTGGATTTAGCAGTAAACT | 68 | A-G | L | 0.06 |
| 2259 | TCCCAAACATATAACTGAACTCCTCA | 26 | C-T | L | 0.2 |
| 5250 | TTCTTCGATAATGGCCCATTTGGGCA | 26 | T-C | H | 0.2 |
| 5263 | (gact)_2_gacGGCTTTTTGCCCAAATGGG | 30 | C-T | L | 0.2 |
| 8869 | (gact)_2_gTTATGAGCGGGCACAGTGATT | 30 | A-G | L | 0.6 |
| 8994 | (gact)_9_gacGCCTACTCATTCAACCAATAGCCCT | 64 | G-A | L | 0.06 |
| 9336 | (gact)_3_gacTTCCACTCCATAACGCTCCTC | 36 | A-G | L | 0.15 |
| 10166 | (gact)_4_gaGGCTACATAGAAAAATCCACCCC | 41 | T-C | L | 0.3 |
| 10211 | (gact)_4_TCCCCCGCCCGCGTCCCTTT | 36 | C-T | L | 0.15 |
| 11140 | (gact)_7_gacCATATTTTATATCTTCTTCGAAACCACACTTAT | 64 | C-T | L | 0.2 |
| 11719 | (gact)_7_gaATTCTCATAATCGCCCACGG | 50 | G-A | L | 0.4 |
| 12308 | (C)_24_ATTGGTCTTAGGCCCCA | 41 | A-G | L | 0.4 |
| 12438 | (gact)_6_gCTAACAAAAAAAACTCATACCCCCA | 50 | T-C | L | 0.15 |
| 12705 | (gact)_3_gaAACATTAATCAGTTCTTCAAATATCTACTCAT | 46 | C-T | L | 0.2 |
| 13101C | (gact)_7_gaGAAGCGGATGAGTAAGAAGATTCC | 54 | A-C | H | 0.2 |
| 13105 | (gact)_4_gaCACTCAAGCACTATAGTTGTAGCAGGA | 45 | A-G | L | 0.2 |
| 14869 | (gact)_13_gacGCTCACTCCTTGGCGCCTGCCT | 77 | G-A | L | 0.2 |
| 14872 | (gact)_12_CTCCTTGGCGCCTGCCTGAT | 68 | C-T | L | 0.4 |
| 15452A | (gact)_8_gGCCCTCGGCTTACTTCTCTTC | 54 | C-A | L | 0.4 |
| 15773 | (gact)_9_gacCCTGAATCGGAGGACAACCA | 59 | G-A | L | 0.1 |
| 15833 | (gact)_9_gacGACAAGTAGCATCCGTACTATACTTCACAACAATC | 74 | C-T | L | 0.2 |
| 15904 | (gact)_10_TTTCATCTCCGGTTTACAAGACTGGTGTATTA | 72 | C-T | H | 0.1 |

**Figure 1.** SNaPshot electropherogram. The three larger pictures show a typical haplogroup V minisequencing profile. The smaller pictures indicate some individual peak (SNP) variant that would identify other R0 branches.


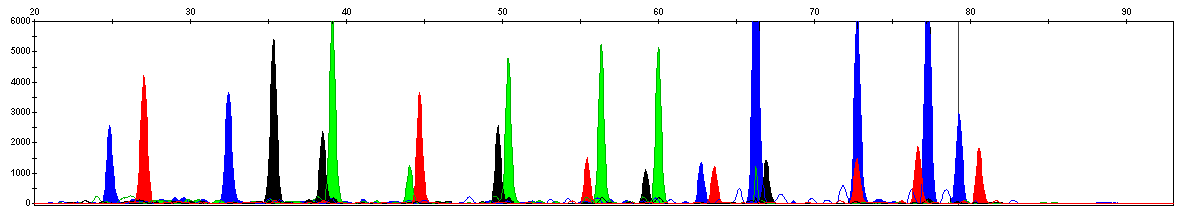


**750**

**2581**

**709**

**2706**

**3796**

**3010**

**4580**

**3847**

**6296**

**6253**

**10810**

**6365**

**12858**

**6776**

**7337**

**12957**

**13759**

**14766**

**14365**

**15218**

**4550**

**14770**

**13708**

**14470**


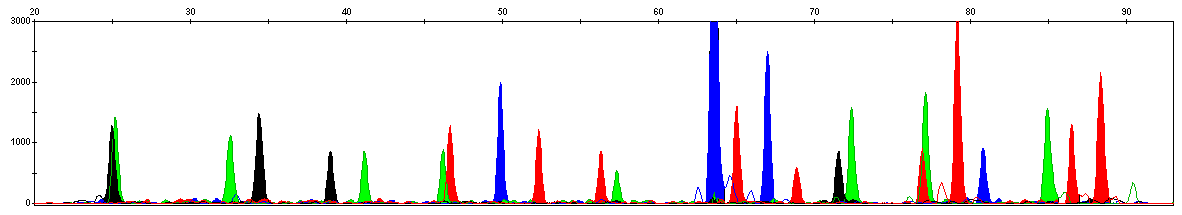


**951**

**4336**

**4745**

**3915**

**3992**

**4310**

**7028**

**4727**

**4769**

**7645**

**8473**

**4793**

**8269**

**9066**

**8592**

**10044**

**10394**

**9150**

**8602**

**9088**

**13404**

**3936**

**8271**

**8598**

**961**


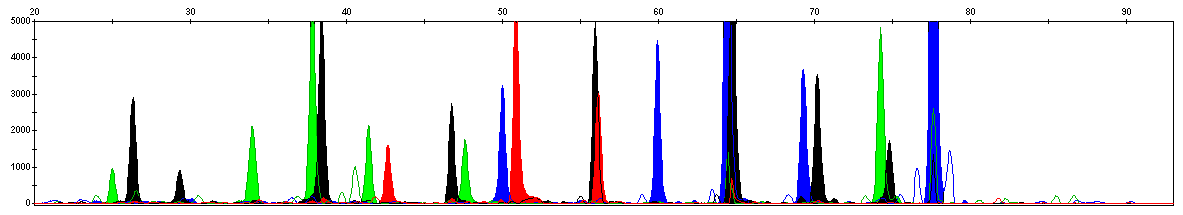


**5250**

**2259**

**5263**

**8869**

**9336**

**10211**

**12308**

**10166**

**12705**

**13105**

**12438**

**11719**

**15452**

**13101**

**15773**

**8994**

**11140**

**1438**

**14872**

**15904**

**15833**

**14869**


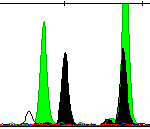


**709**


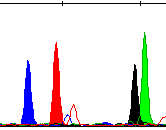


**4580**


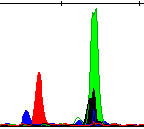


**13759**


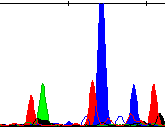


**14365**


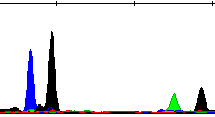


**4336**


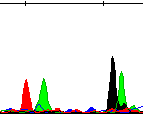


**3992**

**7028**


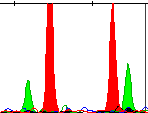


**4769**


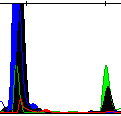


**1438**


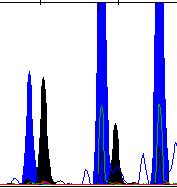


**15904**


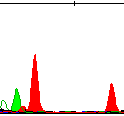


**12705**


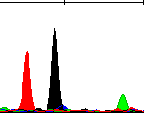


**2259**

**Multiplex 1**

**Multiplex 2**

**Multiplex 3**
