# Supplementary material for: New Population and Phylogenetic Features of the Internal Variation within Mitochondrial DNA Macro-Haplogroup R0
Source: PLoS One. 2009 Apr 2;4(4):e5112. doi: 10.1371/journal.pone.0005112 (PMC2660437; doi:10.1371/journal.pone.0005112)
Supplement: Text S2 — Reconciliation of the nomenclature conflicts in haplogroup R0. (0.14 MB DOC) [file pone.0005112.s002.doc]

**Text S2.**

Below we list some of the nomenclature conflicts observed in the phylogeny of haplogroup R0 and the justification for the nomenclature of new R0 sub-branches. Recently, van Oven and Kayser (2008) have carried out an important effort in updating the worldwide mtDNA phylogeny and nomenclature, including R0. However their updated version does not cover all the necessary changes. In what follows we show the main changes in nomenclature with respect to previous efforts; the full list of changes is however reflected in the phylogeny of Figure 1 and 2 and Table S3.

- As reported in Brandstatter et al. (2006; 2008), T16209C, together with T6365C, is part of the motif of H1a1 (e.g. T16209C is present in all complete genomes from Coble et al. (2004)); note that other recent studies did not consider this position as part of the motif (Roostalu et al. 2007; van Oven and Kayser 2008);
- Similarly, T16263C, together with A9150G, is part of the motif of H1c1; see for instance Coble et al. (2004);
- As indicated in Brandstätter et al. (2006), G8764A is part of the motif of H1c2 coupled with C12858T;
- H1e is defined by Behar et al. (2004), but it is not considered in van Oven and Kayser (2008). There are however at least 17 complete genomes and coding region segments supporting this branch.
- Transitions T8602C T14212C define a new minor branch, here named as H1g, as supported by two coding region segments from Herrnstadt et al. (2002); there is no information concerning the control region;
- We keep the nomenclature of the branch bearing T8473C as H1-8473 (Brandstätter et al. 2006). Position 709 is also highly recurrent, and therefore, we now record two different branches in the tree defined by this transition, namely, H-709 and H1-709;
- We agree with the newly identified branches H2a2b and H2a2b1 of van Oven and Kayser (2008) as these clades are supported by complete genomes EF418606, EU443605, EU444119, and EU719115;
- H2a5 is a new branch identified in the present study (see main text) defined by position A1842G, T4592C, G13708A, C16291T and T16519C;
- H3a is defined by transition T13404C, but also by transition T152C and C16239G (Achilli et al. 2004);
- Variant A73G also characterizes a sub-clade of H3 supported by several complete genomes (e.g. AY495177, AY495180, AY738983 from Coble et al. (2004) and Achilli et al. (2004)), here named as H3d;
- There is a sub-branch of H4a1a carrying transition A73G (H4a1a1; see also (Brandstätter et al. 2006)). The cluster H4a1a defined by (Behar et al. 2008) is here renamed as H4a1b in order to preserved the older nomenclature.
- T961G is part of the motif characterizing haplogroup H11 (supported by seven complete genomes), while H11a is defined only by C8898T. Transition A16293G is not always present in H11 and H11a and therefore it is not considered here as part of the H11a motif;
- We name the whole cluster H13 as in van Oven and Kayser (2008) but without T16519C; however, we here consider that the tip H13a1a1 is defined by only G7337A and T13326C;
- As previously suggested by Roostalu et al. (2007), G11377A is not part of the motif of haplogroup H14 (Achilli et al. 2004);
- H16 embraces an small branch, H16a, defined by G8592A supported by seven complete genomes (e.g. see data from (Coble et al. 2004));
- We keep the nomenclature of Roostalu et al. (2007) for H19, defined by transition G14869A; while the H19 ‘Etruscan’ lineage defined by G16145A A16227G is here renamed as H22;
- There is a new minor branch, H23, defined by transition C10211T (see (Coble et al. 2004) and (Herrnstadt et al. 2002));
- We baptize new clades within HV0a. With respect to the nomenclature provided in Palanichamy et al. (2004), we here define the following sub-clades: a) V1a1 is determined by A227G, T485C, and G709C (supported by data from (Finnilä et al. 2001)), b) V2a1 harbors T9088C and T14793C, and there is a sub-branch V2a1a defined by C5250T, c) V2b is characterized by C14770T, T16519C, and V2b1 has in addition G15773A (see data from (Coble et al. 2004)), d) haplogroup V3 bears variants T4550C, A8347G, A12810G, T13500C, G15346A, and T16519C (see AY495328 and AY495330 in (Coble et al. 2004)).
- We added HV1a1a1a to the tree of HV1, see (Behar et al. 2008; van Oven and Kayser 2008).
- We follow van Oven and Kayser (2008) in that HV1b is defined by T12696C. We add HV1b1 to the tree defined by T2626C C4739T G16274A; see also Behar et al. (2008).
- HV3 in Shen et al. (2004) refers to the same branch reported in Palanichamy et al. (2004) as HV2; therefore, this clade is named here as HV2 although we know now that its motif is T7193C A9336G T11935C C12061T T16217C (van Oven and Kayser 2008).
- We follows van Oven and Kayser (2008) for the nomenclature of HV4 to HV9, which basically modifies the one from Malyarchuk et al. (2008).
- The phylogeny of R0a is complex because alignment problems related to variation (substitutions and indels) around the T-homopolymeric track 57-60, see (Brandstätter et al. 2008). Moreover, there are several nomenclature conflicts in the literature as indicated in the main text of the present study. We here favored the alignment (Table 1) that better fits with the consensus according to what is nowadays found in the literature and GenBank. We have nevertheless to assume that the GenBank data from Abu-Amero et al. (2007) contains several errors: a) sequences DQ904235, DQ904237, and DQ904239 has the transversion T58A; however, their Figure 2 and the literature on control region segments plus other two complete genomes (EU597493, F660971) belonging to R0a1a all carry a transition T58C, b) sequences DQ904238 and DQ904240 bear T59A, but their Figure 2 displays T58C, and finally, c) their three complete genomes belonging to R0a1a (DQ904235, DQ904237, DQ904239) has by mistake a transversion C16355A, but their Figure 2 again shows a transition C16355T; the later is further supported by several dozen of control region segments available in the literature (e.g. (Richards et al. 2000)). A new minor branch defined by T152C A153G G3438A C10728T is named here as R0a2d (supported by complete genomes AY738940 and EF436244).

It is worth mentioning that there are some branches that are not shown in the updated tree of van Oven and Kayser (2008), such as H13a2b, H14a1, H16a, etc. In addition, note also we did not consider here length variation around position 310 and 514-524, as done by others (van Oven and Kayser 2008), because the high mutation rate characterizing these segments. Some other positions are also too unstable (e.g. 152, 16519) and therefore, the phylogeny will need further revision in the near future.

**References**

Abu-Amero KK, González AM, Larruga JM, Bosley TM, Cabrera VM (2007) Eurasian and African mitochondrial DNA influences in the Saudi Arabian population. BMC Evol Biol 7: 32.

Achilli A, Rengo C, Magri C, Battaglia V, Olivieri A et al. (2004) The molecular dissection of mtDNA haplogroup H confirms that the Franco-Cantabrian glacial refuge was a major source for the European gene pool. Am J Hum Genet 75(5): 910-918.

Andrews RM, Kubacka I, Chinnery PF, Lightowlers RN, Turnbull DM et al. (1999) Reanalysis and revision of the Cambridge reference sequence for human mitochondrial DNA. Nat Genet 23: 147.

Behar DM, Hammer MF, Garrigan D, Villems R, Bonne-Tamir B et al. (2004) MtDNA evidence for a genetic bottleneck in the early history of the Ashkenazi Jewish population. Eur J Hum Genet 12(5): 355-364.

Behar DM, Metspalu E, Kivisild T, Rosset S, Tzur S et al. (2008) Counting the founders: the matrilineal genetic ancestry of the Jewish Diaspora. PLoS ONE 3(4): e2062.

Brandstätter A, Salas A, Niederstätter H, Gassner C, Carracedo Á et al. (2006) Dissection of mitochondrial superhaplogroup H using coding region SNPs. Electrophoresis 27(13): 2541-2550.

Brandstätter A, Zimmermann B, Wagner J, Göbel T, Röck A et al. (2008) Timing and deciphering mitochondrial DNA macro-haplogroup R0 variability in Central Europe and Middle East. BMC Evol Biol 4(8): 191.

Coble MD, Just RS, O'Callaghan JE, Letmanyi IH, Peterson CT et al. (2004) Single nucleotide polymorphisms over the entire mtDNA genome that increase the power of forensic testing in Caucasians. Int J Legal Med 118(3): 137-146.

Finnilä S, Lehtonen MS, Majamaa K (2001) Phylogenetic network for European mtDNA. Am J Hum Genet 68: 1475-1484.

Gasparre G, Porcelli AM, Bonora E, Pennisi LF, Toller M et al. (2007) Disruptive mitochondrial DNA mutations in complex I subunits are markers of oncocytic phenotype in thyroid tumors. Proc Natl Acad Sci U S A 104(21): 9001-9006.

Hartmann A, Thieme M, Nanduri LK, Stempfl T, Moehle C et al. (2008) Validation of microarray-based resequencing of 93 worldwide mitochondrial genomes. Hum Mutat.

Herrnstadt C, Elson JL, Fahy E, Preston G, Turnbull DM et al. (2002) Reduced-median-network analysis of complete mitochondrial DNA coding-region sequences from the major African, Asian, and European haplogroups. Am J Hum Genet 70: 1152-1171.

Malyarchuk B, Grzybowski T, Derenko M, Perkova M, Vanecek T et al. (2008) Mitochondrial DNA phylogeny in Eastern and Western Slavs. Mol Biol Evol 25(8): 1651-1658.

Palanichamy Mg, Sun C, Agrawal S, Bandelt H-J, Kong Q-P et al. (2004) Phylogeny of mitochondrial DNA macrohaplogroup N in India, based on complete sequencing: implications for the peopling of South Asia. Am J Hum Genet 75(6): 966-978.

Richards M, Macaulay V, Hickey E, Vega E, Sykes B et al. (2000) Tracing European founder lineages in the Near Eastern mtDNA pool. Am J Hum Genet 67: 1251-1276.

Roostalu U, Kutuev I, Loogväli E-L, Metspalu E, Tambets K et al. (2007) Origin and expansion of haplogroup H, the dominant human mitochondrial DNA lineage in West Eurasia: the Near Eastern and Caucasian perspective. Mol Biol Evol 24(2): 436-448.

Shen P, Lavi T, Kivisild T, Chou V, Sengun D et al. (2004) Reconstruction of patrilineages and matrilineages of Samaritans and other Israeli populations from Y-chromosome and mitochondrial DNA sequence variation. Hum Mutat 24(3): 248-260.

van Oven M, Kayser M (2008) Updated comprehensive phylogenetic tree of global human mitochondrial DNA variation. Hum Mutat in press.

**Table 1**. Alignment of R0 mtDNAs for the sequence range 50-74 (HVS-II).

| **GenBank** | **Reference** | **HG** | **Alignment (50-74)** | **Annotated variants** |
| --- | --- | --- | --- | --- |
| J01415 | [1] | rCRS | TTTGGTATTTT--CGTCTGGGGGGTAT |  |
| EU597493 | [2] | R0 | TTTGGTATCTTT-CGTTTGGGGGGTAT | 58 60+T |
| DQ904236 | [3] | R0a1 | TTTGGTATTTT--CGTTTGGGGGGTAT |  |
| DQ904235 | [3] | R0a1a | TTTGGTATATT--CGTTTGGGGGGTAT | 58A |
| DQ904237 | [3] | R0a1a | TTTGGTATATT--CGTTTGGGGGGTAT | 58A |
| DQ904239 | [3] | R0a1a | TTTGGTATATT--CGTTTGGGGGGTGT | 58A |
| EF660971 | [4] | R0a1a | TTTGGTATCTT--CGTTTGGGGGGTAT | 58 |
| AY713999 | [5] | R0a2 | TTTGGTATTTTT-CGTTTGGGGGGTAT | 60+T |
| DQ904238 | [3] | R0a2 | TTTGGTATTATT-CGTTTGGGGGGTAT | 59A 60+T |
| DQ904240 | [3] | R0a2 | TTTGGTATTTTT-CGTTTGGGGGGTAT | 60+T |
| DQ904241 | [3] | R0a2 | TTTGGTATTATT-CGTTTGGGGGGTAT | 59A 60+T |
| DQ904242 | [3] | R0a2 | TTTGGTATTTTT-CGTTTGGGGGGTAT | 60+T |
| EF660974 | [4] | R0a2 | TTTGGTATCTTT-CGTTTGGGGGGTAT | 58 60+T |
| EF556170 | [6] | R0a2a | TTTGGTATCTTT-CGTTTGGGGGGTAT | 58 60+T |
| EF556172 | [6] | R0a2b | TTTGGTATCTTT-CGTTTGGGGGGTAT | 58 60+T |
| EF556176 | [6] | R0a2c | TTTGGTATCTTTTCGTTTGGGGGGTAT | 58 60+TT |
| AY738940 | [7] | R0a2d | TTTGGTATTTTT-CGTTTGGGGGGTAT | 60+T |
| EF436244 | [8] | R0a2d | TTTGGTATTTTT-CGTTTGGGGGGTAT | 60+T |

[1] Andrews et al. (1999); [2] Hartmann et al. (2008); [3] Abu-Amero et al. (2007); [4] Gasparre et al. (2007); [5] Palanichamy et al. (2004); [6] Behar et al. (2008); [7] Achilli et al. (2004); [8] Family Tree DNA (GenBank).
