# Supplementary material for: New Population and Phylogenetic Features of the Internal Variation within Mitochondrial DNA Macro-Haplogroup R0
Source: PLoS One. 2009 Apr 2;4(4):e5112. doi: 10.1371/journal.pone.0005112 (PMC2660437; doi:10.1371/journal.pone.0005112)
Supplement: Text S3 — Note about the advantages of using minisequencing high throughput SNP genotyping and report of the phylogenetic inconsistencies observed in the data from North Iberia. (0.04 MB DOC) [file pone.0005112.s003.doc]

**Text S3.**

*The minisequencing high throughput SNP genotyping strategy*

Analysis of mtDNA SNPs always supposes a challenge for those laboratories carrying out tedious RFLP analysis of several dozens of coding region SNPs. In addition to the high cost and personal effort related to the genotyping of coding region SNPs, there is also an immanent risk for sample cross-over and contamination due to the number of PCR amplicons involved. In order to overcome this problem, the SNP genotyping carried out in the present study was based on a minisequencing technique which has been extremely useful in many clinical, forensic, and population genetic applications (Quintáns et al. 2004; Álvarez-Iglesias et al. 2007; Álvarez-Iglesias et al. 2008). The minisequencing reactions designed in the present study allow to genotype a total of 71 SNPs in three simple reactions. This method tries to avoid the hierarchical SNP genotyping approach which is the standard procedure in Y-chromosome SNP genotyping studies but also frequently exercised in mtDNA ones. Genotyping the whole set of SNPs in all the samples can also be useful to gain information about site-specific mutation rates and the phylogeny.

Several phylogenetic inconsistencies were observed in the present study (Table S2); some of these inconsistencies involved important diagnostic sites of the mtDNA phylogeny. For instance, G11719A is present in three H* samples (therefore all carry A2706G, C7028T, C14766T); two from Galicia (#C170 and #C203) and one from Catalonia (#AU87). Also interesting is the case of sample #AU74 from Catalonia. This sample belongs to HV (it lacks e.g. G11719A C14766T C16223T) and it carries A2706G and C7028T (it does not belong to haplogroup H). However, this sample carries G4580A which defines haplogroup V but it does not carries the necessary mutations C15904T and T16298C for this haplogroup. Sample #C25 belongs to H6a1 and carries the variant C15452T; note that the transversion C15452A defines haplogroup JT. To our knowledge there is only one record for C15452T, but since it occurs within haplogroup J (Uusimaa et al. 2004), it could just represent a documentation error from the necessary transversion at this position.

*References*

Álvarez-Iglesias V, Jaime JC, Carracedo Á, Salas A (2007) Coding region mitochondrial DNA SNPs: targeting East Asian and Native American haplogroups. Forensic Sci Int: Genet 1: 44-55.

Álvarez-Iglesias V, Barros F, Carracedo Á, Salas A (2008) Minisequencing mitochondrial DNA pathogenic mutations. BMC Med Genet 9: 26.

Quintáns B, Álvarez-Iglesias V, Salas A, Phillips C, Lareu MV et al. (2004) Typing of mitochondrial DNA coding region SNPs of forensic and anthropological interest using SNaPshot minisequencing. Forensic Sci Int 140(2-3): 251-257.

Uusimaa J, Finnilä S, Remes AM, Rantala H, Vainionpää L et al. (2004) Molecular epidemiology of childhood mitochondrial encephalomyopathies in a Finnish population: sequence analysis of entire mtDNA of 17 children reveals heteroplasmic mutations in tRNAArg, tRNAGlu, and tRNALeu(UUR) genes. Pediatrics 114(2): 443-450.
