# Supplementary figures and images for: New Population and Phylogenetic Features of the Internal Variation within Mitochondrial DNA Macro-Haplogroup R0
Source: PLoS One. 2009 Apr 2;4(4):e5112. doi: 10.1371/journal.pone.0005112 (PMC2660437; doi:10.1371/journal.pone.0005112)

## Slide 1
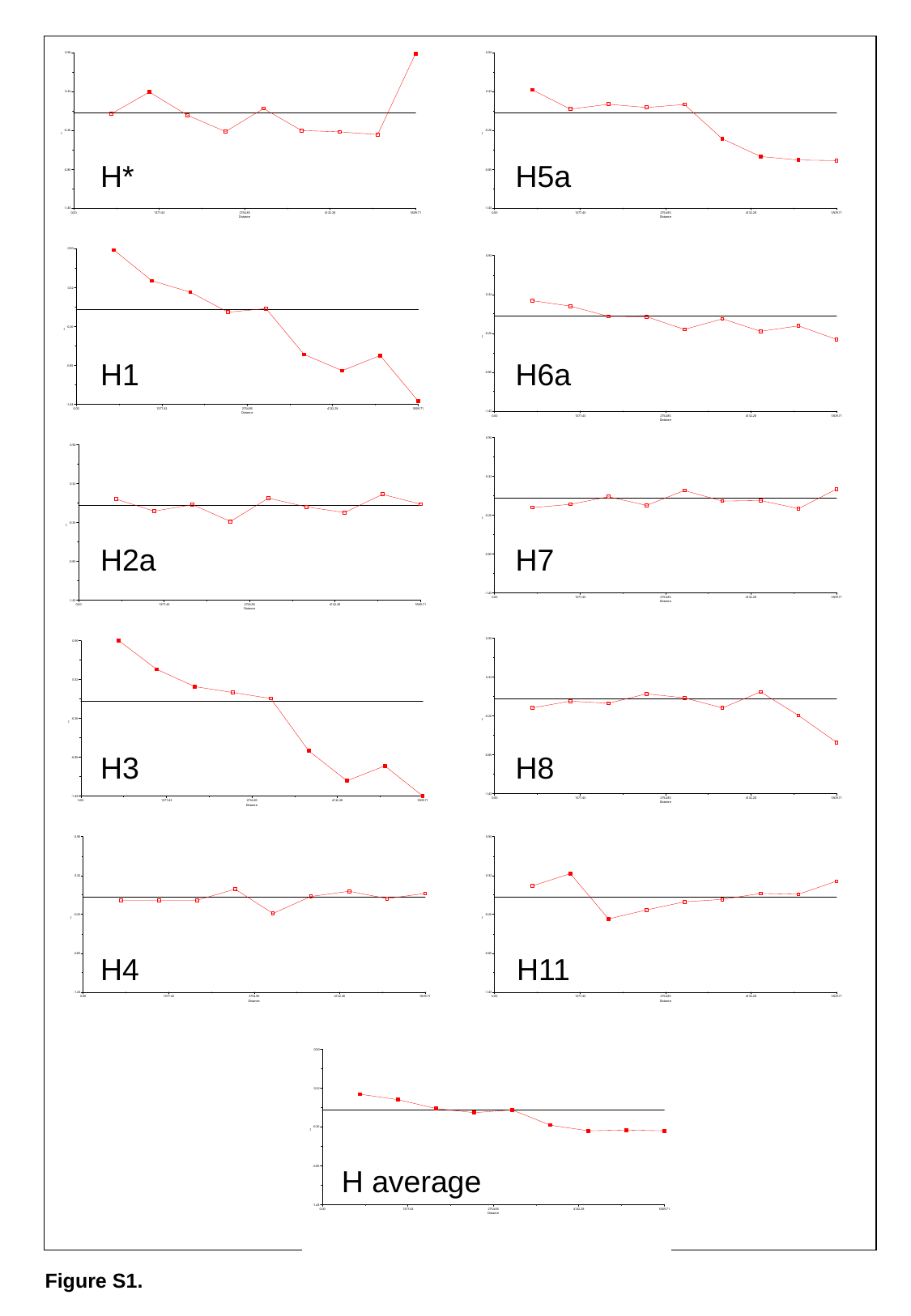

H*
H5a
H1
H6a
H2a
H7
H3
H8
H4
H11
H average
Figure S1.

Supplement: Figure S1 — Autocorrelograms for the most frequent R0 sub-clades observed in North Iberia. (0.12 MB PPT) [file pone.0005112.s007.ppt]
